# The not-so-infinite malleability of RNA viruses: Viral and cellular determinants of RNA virus mutation rates

**DOI:** 10.1371/journal.ppat.1006254

**Published:** 2017-04-27

**Authors:** Everett Clinton Smith

**Affiliations:** Department of Biology, Sewanee: The University of the South, Sewanee, Tennessee, United States of America; University of Kentucky, UNITED STATES

## Introduction

Highly mutable, infinitely malleable, and all-powerful: this is often the underlying assumption for how spontaneous mutations fuel RNA virus adaptation. Though essential for adaptation, mutations within RNA virus genomes can exact significant fitness costs. Without the capacity to detect and repair mismatched or damaged nucleotides, viral RNA genomes are prone to mutations introduced by mechanisms intrinsic and extrinsic to viral replication. However, large population size, complementation, cellular chaperones, and recombination can buffer viral populations against deleterious and lethal mutations. As such, viral replication is a rapid, tenuous dance between the generation of sufficient genetic diversity on which natural selection can act and the production of less-fit variants. The purpose of this article is not to describe how RNA viruses evolve. Rather, it aims to provide an introduction to some of the mechanisms by which mutations arise during RNA virus replication, as viral mutation rates are the ultimate source of genetic diversity.

## What is a mutation rate?

**Fig 1:**
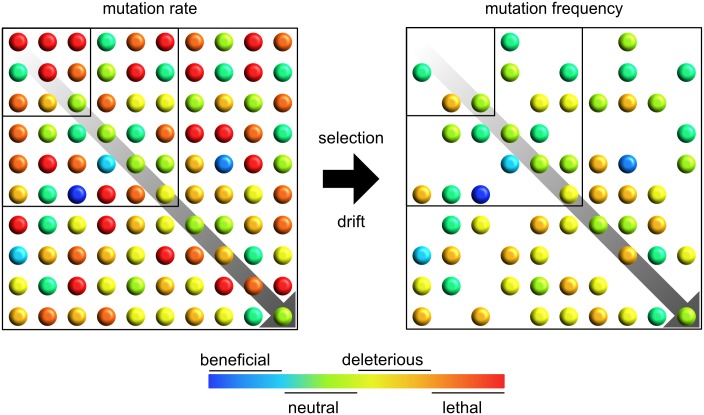
Large viral population sizes buffer against deleterious and lethal mutations. Mutations generated via virus- and cell-dependent mechanisms can differentially impact viral fitness. Viruses are depicted as colored spheres corresponding to the presence of beneficial, neutral, deleterious, or lethal mutations. Population size is depicted by the diagonal arrow and increasing square size. High mutation rates result in a phenotypically diverse viral population (“mutation rate” panel), but the frequency at which these variants appear in a population (“mutation frequency” panel) depends upon genetic drift and natural selection. As such, large population sizes are critical for buffering the viral population against the effects of deleterious and lethal mutations. The ratios of beneficial, neutral, deleterious, and lethal mutations are based on data from vesicular stomatitis virus (VSV) [1].

Storage and transmission of genetic information depends upon the correct formation of hydrogen bonds between nucleobases. Mutations arise when mismatches are introduced during RNA virus replication or as a result of postreplicative base modification. Host RNA-modifying enzymes and nitration or oxidation of nucleobases can alter hydrogen bonding and increase the probability of point mutations during subsequent rounds of replication. A mutation rate describes the rate (not frequency) at which spontaneous mutations arise during a single infection and reflects both cell- and virus-dependent mechanisms. Because most mutations are likely lethal or deleterious [1,2], natural selection and genetic drift significantly impact the observed frequency of mutations within a population (Fig 1). Excluding viroids, RNA viruses replicate with the highest known mutation rates, which are estimated to range between 10^−6^ and 10^−4^ substitutions per nucleotide per cell infection [3]. Such high mutation rates enable viral populations to rapidly generate genetic diversity and thus a multitude of phenotypes on which adaptation by natural selection can occur. However, high mutation rates alone are not sufficient to drive viral adaptation. As mentioned above, many mutations are deleterious and decrease viral fitness [1,2]. How a given mutation affects viral fitness is also dependent upon other characteristics of RNA virus populations, such as population size, genome size, and genome complexity.

## Why is RNA virus replication more error-prone?

One of the main contributors to viral mutation rates is the intrinsic fidelity of the viral RNA-dependent RNA polymerase (RdRp) or retroviral reverse transcriptase (RT). Replication fidelity specifically describes how accurately an RNA or DNA genome is copied relative to the template strand. Cellular DNA replication fidelity can be simplified to three major conceptual steps: (i) nucleotide selection and extension by the polymerase; (ii) removal of mismatched nucleotides by polymerase-associated 3ʹ-to-5ʹ exonuclease (proofreading) activity; and (iii) postreplicative repair of mismatched or damaged nucleotides. RNA viruses lack postreplicative repair, and the key difference between the intrinsic fidelity of the viral RdRp or RT and most replicative cellular DNA polymerases is the lack of associated proofreading activity [4]. Error-prone DNA polymerases do exist, such as Y-family DNA polymerases, but these are mainly involved in translesion synthesis [5]. Kinetic studies of the poliovirus RdRp (3D^pol^) demonstrate that RdRp replication fidelity is similar to DNA polymerases lacking exoribonuclease (ExoN) activity [6]. Hence, RNA virus replication is error prone due to the lack of proofreading activity, not because of an intrinsically lower fidelity polymerase. The one exception to this rule are nidoviruses within the families Coronaviridae, Roniviridae, and Mesoniviridae, which encode proofreading 3’-to-5’ exoribonuclease activity within a protein distinct from the viral RdRp. Despite the lack of bona fide proofreading activity, removal of nucleotides from a nascent RNA strand during viral replication is not unprecedented. Nucleoside analogs are a class of antiviral compounds that mimic endogenous nucleotides upon phosphorylation and can be removed by the RdRp via pyrophosphate- or nucleotide-dependent pyrophosphorolysis [7]. Whether and to what extent the kinetic battle between pyrophosphorolysis and polymerization of endogenous cellular nucleotides impacts viral mutation rates is unknown.

## How can replication fidelity be altered?

The intrinsic fidelity of viral RdRps is governed by multiple biochemical and biophysical checkpoints, such as conformational changes, mediated by amino acids proximal to and distal from the RdRp active site (reviewed in [8]). Studies with poliovirus 3D^pol^ provided the first descriptions of high- and low-fidelity RdRp variants [9,10]. The high fidelity G64S 3D^pol^ variant was isolated following passage in the presence of the antiviral nucleoside analog ribavirin, and resulted in a 3-fold increase in fidelity [9]. Though nonrecoverable as a recombinant virus, a N297E substitution within 3D^pol^ decreased fidelity by 14-fold and provided early evidence for the existence of a lower limit for replication fidelity [10]. Subsequent adaptive passage experiments and structure-guided mutagenesis have identified numerous high- and low-fidelity RdRps across multiple virus families (see [11] for a review). These RdRp variants most commonly have a 2-to-5-fold increase or decrease in replication fidelity and often are attenuated—to varying degrees—in animal models of disease [11]. RdRps with a greater-than 3-fold reduction in fidelity are typically nonrecoverable, suggesting a lower limit for changes in replication fidelity. No upperlimit for replication fidelity has been reported, but even a 3-fold increase in poliovirus replication fidelity is attenuating due to restricted population diversity [12]. Though the vast majority of altered-fidelity variants are RdRp mutants, other viral proteins are implicated in replication fidelity. Inactivation of coronavirus ExoN activity results in a mutator phenotype [13], and mutations within coronavirus nonstructural protein 10 increase coronavirus sensitivity to base and nucleoside analogs, suggestive of low fidelity [14]. Additional examples such as the T248I mutation within the West Nile virus methyltransferase and the G641D mutation within chikungunya virus nonstructural protein 2 (nsP2) can decrease and increase fidelity, respectively [15,16]. Because viral RNA replication ultimately requires protein complexes, mutational disruption of viral protein–protein interactions or alterations in replicase protein stoichiometry could all presumably alter fidelity.

## Which cellular factors contribute to viral mutation rates?

Because viruses are dependent upon the host cell, replication is inexorably linked to the cellular microenvironment. As such, viral mutation rates reflect multiple events intrinsic and extrinsic to viral replication. Vesicular stomatitis virus (VSV) replicates with a similar mutation rate across multiple mammalian cell lines but has a lower mutation rate during replication in insect cells [17]. Cucumber mosaic virus exhibits different mutational spectra following inoculation of different plant hosts, suggesting that disparate intracellular conditions can modulate replicase fidelity and, presumably, the mutation rate [18]. A recent study with poliovirus indicates that the RdRp is not the exclusive source of genetic variation within an RNA virus population [19]; however, the exact mechanisms underlying these variations are unknown. Intracellular nucleotide concentration, oxidation or nitration of nucleobases, and the presence of host RNA-modifying enzymes are all potential mechanisms by which mutations could be introduced (see [20] for an extensive review).

Altered concentrations of host ribonucleotide triphosphate (rNTP) pools could influence viral mutation rates, as viruses exclusively use host nucleotides. Inhibitors of enzymes critical for de novo purine and pyrimidine biosynthesis, such as mycophenolic acid and brequinar, can exert a potent antiviral effect during RNA virus replication [15,21]. Low-fidelity RNA viruses are more sensitive to such inhibitors [22], whereas high fidelity RNA viruses are more resistant [15]. Intracellular rNTP levels were not examined in either case. These compounds have pleiotropic effects on both viral and cellular enzymes, making it difficult to identify the precise mechanism(s) through which antiviral activity is exerted.

Both reactive oxygen and nitrogen species (ROS and RNS, respectively) can be generated during viral infection and damage all classes of biomolecules, including nucleobases. Watson-Crick base pairing is predicated on hydrogen bonding, and the oxidation or nitration of cellular nucleobases effectively modulates hydrogen bonding capacity. The resulting nucleobases, such as 8-oxoguanine or 8-nitroguanine, can be mutagenic. Nitric oxide (NO), a physiologically relevant RNS produced during viral infection, is mutagenic during Sendai virus replication but is not antiviral [23]. Similar to RNS-induced mutagenesis, virus- and ethanol-induced ROS increase viral mutations during hepatitis C replication [24]. Oxidation or nitration of rNTP pools could impact viral mutation rates across multiple families of RNA viruses. Whether this promotes viral adaptation and pathogenesis or is exclusively antiviral remains unclear.

Host-encoded protein families such as apolipoprotein B mRNA-editing enzyme, catalytic polypeptide-like (APOBEC) and adenosine deaminase acting on RNA (ADAR) can modify viral nucleic acid. APOBEC3G is packaged into HIV-1 virions and deaminates cytosine bases to uracil within viral complementary DNA (cDNA) [25]. This results in G-to-A base substitutions and generates 98% of mutations within HIV-1 in vivo [26]. Though predominantly antiviral, APOBEC-mediated editing could also contribute to pathogenesis in situations in which edited viruses are viable. Whereas APOBEC proteins modify DNA, ADAR proteins modify genomic RNA or viral transcripts containing double-stranded RNA (dsRNA) structures. ADAR deaminates adenine bases to inosine, resulting in A-to-G base substitutions, and occurs across RNA virus families resulting in either pro- or antiviral effects [27]. In summary, multiple mechanisms can impact viral mutation rates and whether and to what extent each is pro- or antiviral is an important focus of future research.

## Why is understanding viral mutation rates important?

Constant adaptive evolution is essential for RNA viruses to overcome intrinsic, innate, and adaptive host immunity, antiviral therapeutics, and for intra- and inter-host transmission. As the ultimate source of all genetic variation, mutations are key to this adaptive process. Without this phenotypic diversity, adaptation via natural selection cannot occur. Elucidating how spontaneous mutations are generated during viral replication is critical for understanding virus evolution. The capacity to generate RNA viruses with altered fidelity could yield new paradigms for antiviral treatment and live-attenuated vaccine development, assuming sufficient levels of attenuation can be achieved. Most of these studies are based on mutation frequency assays, highlighting the need to determine mutation rates across virus families. Beyond these human health applications, RNA viruses are highly tractable systems that could be used to answer fundamental questions about evolutionary processes in cellular organisms.
